# Lipedema-Related Outcomes During Gestrinone Treatment for Estrogen-Dependent Gynecological Disorders: A Real-World Retrospective Case Series

**DOI:** 10.7759/cureus.116856

**Published:** 2026-09-24

**Authors:** Paula Frederichi de Souza

**Affiliations:** 1 Scientific Department, Educational Organization in Lipedema (ABRALIPRO), São Paulo, BRA

**Keywords:** endometriosis, genital diseases, gestrinone, hormone replacement therapy, lipedema

## Abstract

Background: The clinical course of lipedema in women receiving hormonal treatment for concomitant estrogen-dependent gynecological disorders remains insufficiently characterized. This study aimed to describe gynecological and lipedema-related manifestations during routine follow-up in women receiving subcutaneous gestrinone implants for gynecological indications.

Methods: This retrospective, descriptive case series included 50 women with stage II or III lipedema and concomitant estrogen-dependent gynecological disorders treated between May 2024 and May 2025 at a specialized multidisciplinary outpatient clinic in São Paulo, Brazil. Participants received two biodegradable subcutaneous gestrinone implants (35 mg and 50 mg; total dose: 85 mg) exclusively for gynecological indications. Clinical outcomes were extracted retrospectively from medical records and analyzed descriptively. Improvement proportions were calculated using the number of patients presenting each manifestation at baseline as the denominator. Outcomes were based on patient reports and qualitative clinical assessments without standardized instruments.

Results: Among gynecological manifestations, improvement was documented in 43 of 50 patients with menstrual pain (86%), six of 13 with menorrhagia (46.2%), and two of 50 with premenstrual symptoms (4%). Among lipedema-related manifestations, improvement was documented in 35 of 39 patients with edema (89.7%) and 33 of 41 with spontaneous pain (80.5%). Qualitative improvement in skin appearance and clinically assessed tissue fibrosis was documented in 31 of 50 (62%) and 28 of 50 (56%) patients, respectively. Overall clinical status was reported as improved by 42 patients (84%), stable by six (12%), and worsened by two (4%).

Conclusions: Improvements in pain and edema, alongside qualitative changes in skin appearance and clinically assessed tissue fibrosis, were documented during routine follow-up. Given the retrospective design, absence of a comparison group, and nonstandardized assessments, these observations cannot be attributed to gestrinone or interpreted as evidence of its efficacy for lipedema. Prospective controlled studies using standardized outcome measures are warranted.

## Introduction

Hormonal factors have been investigated in the pathophysiology of lipedema due to its predominance in women and the reported onset or progression of the disease during puberty, pregnancy, and menopause [1-3]. Histological and molecular findings indicate alterations in adipocyte physiology, extracellular matrix remodeling, inflammatory processes, and microvascular and lymphatic function in affected tissue [4-6]. Experimental studies have identified changes in estrogen receptor expression and enzymes involved in estrogen metabolism in adipose-derived cells from women with lipedema. Although these findings support the investigation of hormonal pathways in the disease, current evidence remains insufficient to define the role of estrogen signaling in the development and progression of lipedema [7,8].

The involvement of estrogen-related pathways is also relevant when considering the coexistence of lipedema with hormone-dependent gynecological disorders. Endometriosis, adenomyosis, and uterine fibroids have been reported among women with lipedema, although the clinical significance of these associations remains to be established [9,10]. These gynecological conditions are characterized by alterations in local steroid regulation, including estrogen synthesis and metabolism, progesterone responsiveness, and hormone-mediated inflammatory processes [11-13]. Some of these pathways have also been investigated in lipedema, suggesting potential biological connections between these conditions [8]. However, their coexistence does not establish a shared pathophysiological mechanism or a causal relationship.

Gestrinone is a synthetic steroid with antiestrogenic, antiprogestogenic, androgenic, and antigonadotropic activity that has been investigated for the treatment of endometriosis [14-16]. Its pharmacological effects involve interactions with steroid hormone receptors and suppression of ovarian steroidogenesis, thereby modifying hormonal stimulation in responsive tissues [14]. Clinical evidence supports its effects on endometriosis-associated symptoms, particularly dysmenorrhea and pelvic pain [15-17]. These pharmacological properties provide a clinical context for documenting lipedema-related manifestations in women receiving gestrinone for gynecological indications, without implying a therapeutic effect on lipedema.

Women with lipedema and concomitant estrogen-dependent gynecological disorders represent a clinically relevant population in which changes in both conditions may be documented during hormonal treatment. Observations obtained from routine clinical records may provide preliminary information to guide prospective studies investigating the relationship between hormonal treatment and lipedema-related outcomes. However, such observations cannot establish whether documented changes are attributable to hormonal treatment or other factors associated with routine clinical care.

Therefore, this retrospective case series aimed to describe the documented changes in gynecological and lipedema-related manifestations during approximately 20 weeks of routine clinical follow-up in women with stage II or III lipedema who received subcutaneous gestrinone implants for concomitant estrogen-dependent gynecological disorders. The study was descriptive and exploratory and was not designed to evaluate treatment efficacy or establish causality.

## Materials and methods

Study design

This retrospective, descriptive case series was based on the review of medical records of women with a clinical diagnosis of lipedema and concomitant estrogen-dependent gynecological disorders who received subcutaneous gestrinone implants as part of routine gynecological care. Patients were treated between May 2024 and May 2025 at Clínica Integrada Dra. Paula Frederichi, a specialized multidisciplinary outpatient clinic in São Paulo, Brazil. Clinical follow-up was conducted according to routine medical practice for approximately 20 weeks after implantation. Data were retrospectively extracted from medical records between February and April 2026.

The study was approved by the Research Ethics Committee of Mackenzie Presbyterian University (approval number: 8.052.687; CAAE: 94490425.4.0000.0084). The investigation consisted exclusively of a retrospective analysis of secondary data obtained from medical records. No direct patient contact or additional procedures were performed for research purposes.

Participants and clinical context

A total of 50 medical records from women with stage II or III lipedema and concomitant estrogen-dependent gynecological disorders were included in the analysis. The cohort comprised 37 women diagnosed with endometriosis and 13 with uterine fibroids.

The diagnosis of lipedema was established by the treating physician through clinical assessment, including medical history and physical examination, based on the characteristic disproportionate and predominantly symmetrical distribution of adipose tissue in the extremities, associated with symptoms such as pain or tenderness and relative sparing of the hands and feet [2]. Disease staging was determined according to the morphological characteristics of the affected tissues. Stage II was characterized by an irregular skin surface with dimpling and palpable subcutaneous nodules, whereas stage III was characterized by more pronounced tissue enlargement, larger nodules, lobular deformities, and overhanging skin folds.

The 50 medical records were selected from a larger pool of patients treated during the study period based on the availability of sufficient clinical information to evaluate the outcomes of interest. Eligible participants were women with a clinical diagnosis of stage II or III lipedema, a documented gynecological indication for gestrinone treatment, and available clinical follow-up information after implantation. Records with insufficient information for outcome assessment were excluded. Therefore, the study population represented a selected sample based on data completeness rather than a consecutive sample of all potentially eligible patients. Gestrinone was prescribed exclusively for gynecological indications and not as a treatment for lipedema. 

Gestrinone implant therapy

As part of routine gynecological management, participants received two biodegradable subcutaneous gestrinone implants in the upper gluteal region, containing 35 mg and 50 mg, respectively, for a total dose of 85 mg. The implants were prepared by Biòs Farmacêutica (Florianópolis, Santa Catarina, Brazil) and inserted by a trained physician.

The 85-mg regimen was selected according to the treating physician's clinical judgment, with amenorrhea as an intended therapeutic goal for the underlying gynecological disorders. This decision was informed by previous clinical observations of menstrual suppression with lower gestrinone doses in women with lipedema, rather than a formal dose-response evaluation.

According to the manufacturer's technical specifications, the 35-mg implant has an estimated daily release of 0.2-0.3 mg over approximately 4-5 months, whereas the 50-mg implant has an estimated daily release of 0.3-0.4 mg over approximately 5-6 months. These values represent manufacturer-provided estimates; individual drug release kinetics and systemic gestrinone exposure were not directly assessed in this study.

Outcome measures

Clinical outcomes documented during routine follow-up consultations were retrospectively extracted from the medical records. Gynecological outcomes included changes in menstrual pain, menorrhagia, and premenstrual symptoms, whereas lipedema-related outcomes included spontaneous pain, edema, skin appearance, and tissue fibrosis.

All clinical assessments were performed by a single physician throughout the follow-up period. Changes in pain and gynecological symptoms were based on patient-reported information documented in the medical records, whereas changes in edema, skin appearance, and tissue fibrosis were based on qualitative clinical assessments. Standardized clinical photographs, when available, were used to support the assessment of tissue appearance. Overall clinical status was based on patient-reported information and categorized as improved, stable, or worsened. No validated outcome assessment instruments or predefined quantitative thresholds for improvement were used.

Data analysis

Following data extraction and tabulation, a descriptive analysis was performed. Demographic and clinical characteristics were summarized using absolute frequencies (n), percentages (%), and mean±standard deviation, as appropriate.

The prevalence of each gynecological and lipedema-related manifestation at baseline was determined from the medical records. Improvement percentages were calculated using the number of patients presenting each manifestation at baseline as the denominator and the number of patients with documented improvement during follow-up as the numerator.

Overall clinical status was categorized as improved, stable, or worsened, with percentages calculated using the total study population (N=50). No inferential statistical analyses were performed.

## Results

The study included 50 women with stage II or III lipedema and concomitant estrogen-dependent gynecological disorders. The mean age was 40.1±10.0 years, and the mean body mass index (BMI) was 27.9±4.4 kg/m². Of the participants, 37 (74%) had endometriosis, and 13 (26%) had uterine fibroids. The demographic and clinical characteristics of the study population are summarized in Table 1.

**Table 1:** Demographic and clinical characteristics of the study population (n=50)

Characteristic	Value
Age, years	40.1±10.0
BMI, kg/m²	27.9±4.4
Lipedema stage, n (%)	
Stage II	39 (78%)
Stage III	11 (22%)
Concomitant gynecological disorder, n (%)	
Endometriosis	37 (74%)
Uterine fibroids	13 (26%)

Changes in gynecological and lipedema-related manifestations were documented during routine clinical follow-up (Figure 1). Among gynecological manifestations, menstrual pain was present in 50 patients at baseline, of whom 43 (86%) had documented improvement. Menorrhagia was initially present in 13 patients, with improvement documented in six (46.2%). Premenstrual symptoms were reported by 50 patients at baseline, of whom two (4%) experienced improvement.

**Figure 1:**
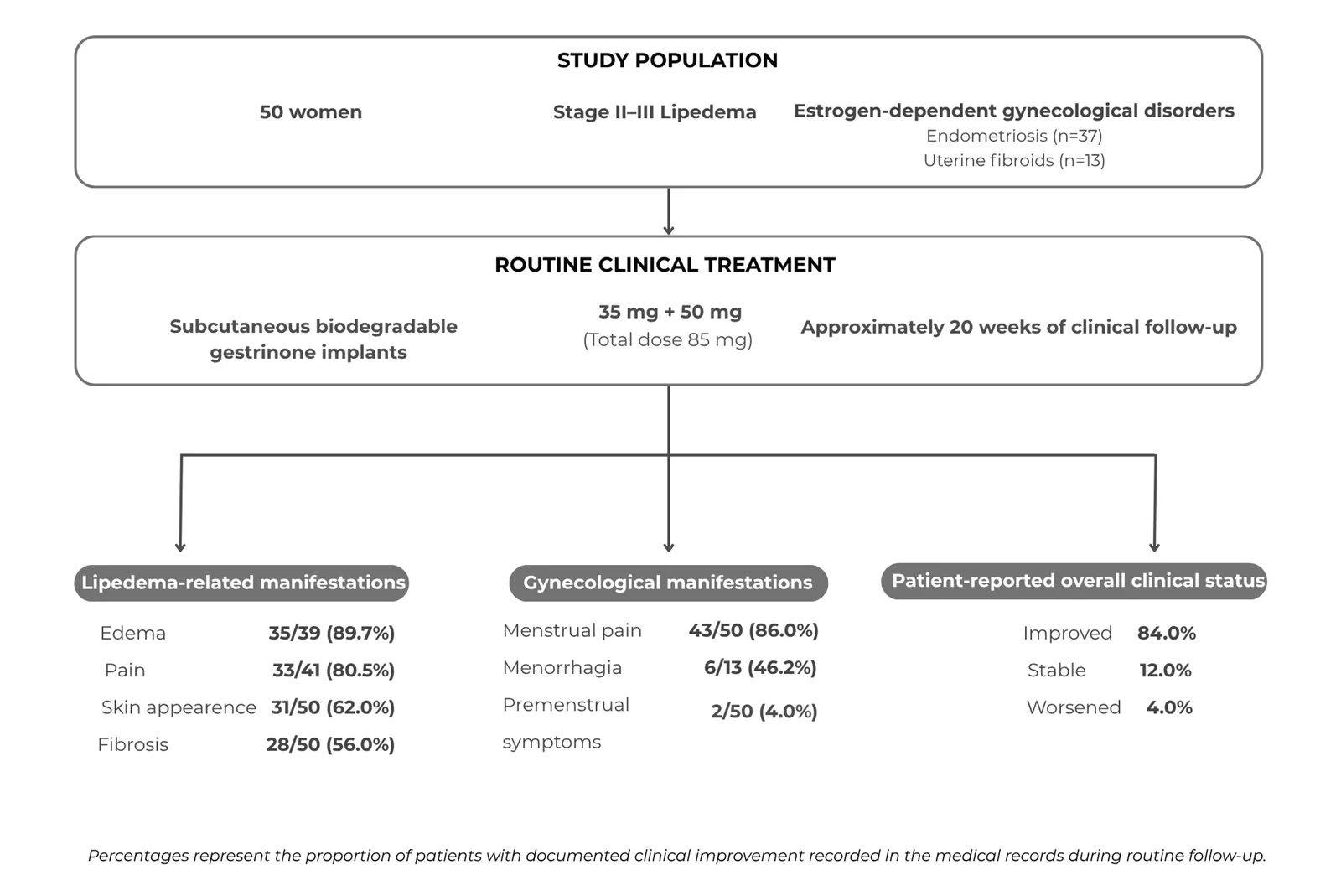
Study overview and main clinical outcomes Schematic summary of the study population, routine clinical management with subcutaneous gestrinone implants, and the main gynecological, lipedema-related, and patient-reported clinical outcomes documented during routine follow-up. The figure was created by the author manually using Canva (Canva Pty Ltd., Sydney, Australia).

Among lipedema-related manifestations, edema was present in 39 patients at baseline, with improvement documented in 35 (89.7%). Spontaneous pain was initially reported by 41 patients, of whom 33 (80.5%) experienced improvement. Changes in skin appearance and tissue fibrosis were documented in 31 (62%) and 28 (56%) patients, respectively, among the 50 patients presenting each manifestation at baseline.

Regarding overall clinical status, 42 (84%) patients reported improvement during follow-up, whereas six (12%) reported stable symptoms, and two (4%) reported worsening of their overall clinical condition.

## Discussion

In this retrospective case series of women with lipedema and concomitant estrogen-dependent gynecological disorders, improvements in gynecological and lipedema-related manifestations were documented during approximately 20 weeks of routine clinical follow-up. Menstrual pain showed the highest proportion of documented improvement among gynecological manifestations, whereas edema and spontaneous pain showed the highest proportions among lipedema-related manifestations. Most participants also reported improvement in their overall clinical status. These findings describe the clinical course of a selected patient population rather than demonstrating a therapeutic effect of gestrinone on lipedema.

The coexistence of lipedema and estrogen-dependent gynecological disorders provides a relevant context for examining clinical changes during hormonal treatment. Sex steroids participate in the regulation of adipocyte function, inflammatory activity, vascular processes, and extracellular matrix remodeling [15-17]. These biological processes are relevant to lipedema, in which alterations involving adipose tissue, inflammation, microvascular function, and extracellular matrix organization have been implicated. Nevertheless, biological plausibility does not establish that hormonal modulation contributed to the clinical changes documented in this cohort.

Pain and edema are prominent manifestations of lipedema and may involve multiple interacting mechanisms. Objective sensory assessments have demonstrated increased mechanical sensitivity in affected regions, together with alterations in dermal neuronal structures and nociceptive mediators [18]. Increased sodium content has also been demonstrated in the skin and subcutaneous adipose tissue of women with lipedema, with reported associations between tissue sodium accumulation, pain intensity, and disease stage [19,20]. These findings suggest that pain and edema occur within a complex tissue environment extending beyond adipose tissue volume alone. Although both manifestations showed documented improvement in the present cohort, spontaneous pain was assessed through patient reports, whereas edema was evaluated qualitatively during routine clinical consultations. Without standardized quantitative measurements, the magnitude and mechanisms of these changes remain undetermined.

Changes in skin appearance and tissue fibrosis were also documented during follow-up. These observations can be considered in the context of structural abnormalities previously described in lipedema, including increased interstitial collagen deposition, adipocyte hypertrophy, and fibrosis in affected adipose tissue [21-23]. However, the present assessments were qualitative and were not supported by histological examination, imaging-based measurements, or quantitative biomechanical testing. Therefore, the recorded changes should be interpreted as clinical observations rather than evidence of reduced tissue fibrosis or structural remodeling.

The pharmacological properties of gestrinone provide additional biological context. Gestrinone interacts with estrogen, progesterone, and androgen receptors, and studies involving human endometrium have demonstrated changes in steroid receptor concentrations and steroid-metabolizing enzyme activity during treatment [24,25]. However, findings obtained in reproductive tissues cannot be extrapolated directly to subcutaneous adipose tissue in women with lipedema. Furthermore, the individual release kinetics of the implants and systemic gestrinone exposure were not measured, precluding examination of potential relationships between drug exposure and the documented clinical outcomes.

The available evidence must also be considered when interpreting these observations. A recent systematic review identified no eligible clinical studies specifically evaluating gestrinone as a treatment for lipedema in its literature search through July 2025 [26]. The present study addressed a different clinical context: gestrinone was prescribed for concomitant gynecological disorders, and changes in lipedema-related manifestations were documented retrospectively. Accordingly, this case series provides exploratory clinical observations but does not establish the efficacy of gestrinone for lipedema.

The observed clinical course must also be interpreted within the context of routine multidisciplinary management. Conservative approaches, including compression, physical activity, and nutritional strategies, may influence pain, swelling, and other manifestations of lipedema [27,28]. Because the study was conducted in a multidisciplinary outpatient setting, concomitant treatments and changes in clinical management represent potential sources of confounding. The available retrospective documentation does not allow their individual contributions to be distinguished from those of hormonal treatment or the natural variability of symptoms over time.

Several methodological limitations warrant consideration. The retrospective and descriptive design, relatively small sample size, absence of a comparison group, and approximately 20-week follow-up limit the interpretation and generalizability of the findings. Medical records were selected according to the availability of sufficient clinical information rather than through consecutive inclusion, introducing the possibility of selection bias. Although improvement proportions were calculated using the number of patients presenting each manifestation at baseline, the available records did not provide standardized measures of baseline symptom severity or predefined quantitative thresholds for improvement. Outcomes relied on patient reports and qualitative clinical assessments rather than validated instruments or objective measurements. Assessments were performed by a single physician, providing consistency in clinical documentation but not eliminating potential observer bias. In addition, concomitant care was not controlled, and individual systemic gestrinone exposure was not assessed. These limitations prevent causal attribution of the documented changes to gestrinone.

Despite these limitations, the simultaneous documentation of gynecological and lipedema-related manifestations during the same follow-up period identifies clinical outcomes that warrant further investigation. Prospective controlled studies incorporating clearly defined eligibility criteria, standardized outcome measures, objective assessments of tissue characteristics, systematic documentation of concomitant treatments and adverse events, and appropriate comparison groups are needed to investigate the clinical course of lipedema in women receiving hormonal treatment for gynecological indications.

## Conclusions

This case series documented improvements in spontaneous pain and edema, alongside qualitative changes in skin appearance and clinically assessed tissue fibrosis, among women receiving gestrinone for gynecological indications. These observations are descriptive and do not establish a therapeutic effect of gestrinone on lipedema. Prospective controlled studies with standardized outcome measures are needed to investigate these clinical findings.
